# Sleep Architecture in Patients With Primary Snoring and Obstructive Sleep Apnea

**DOI:** 10.29252/NIRP.BCN.9.2.147

**Published:** 2018

**Authors:** Kaveh Shahveisi, Amir Jalali, Mohammad Raman Moloudi, Shahla Moradi, Azad Maroufi, Habibolah Khazaie

**Affiliations:** 1. Sleep Disorders Research Center, Kermanshah University of Medical Sciences, Kermanshah, Iran.; 2. Department of Psychiatric Nursing, Faculty of Nursing & Midwifery, Kermanshah University of Medical Sciences, Kermanshah, Iran.; 3. Substance Abuse Prevention Research Center, Kermanshah University of Medical Sciences, Kermanshah, Iran.; 4. Neurosciences Research Center, Kurdistan University of Medical Sciences, Sanandaj, Iran.

**Keywords:** Obstructive sleep apnea, Primary snoring, Sleep architecture, Polysomnography

## Abstract

**Introduction:**

This study aimed to investigate sleep architecture in patients with primary snoring and obstructive sleep apnea.

**Methods:**

In this study, we analyzed polysomnographic data of 391 clients who referred to Sleep Disorders Research Center (SDRS). These people were classified into three groups based on their Apnea-Hypopnea Index (AHI) and snoring; control, Primary Snoring (PS), and Obstructive Sleep Apnea (OSA) group. Sleep architecture variables were then assessed in all groups.

**Results:**

The results of this study indicated a decrease in deep sleep or Slow Waves Sleep (SWS) and increase in light sleep or stage 1 of non-REM sleep (N1) in OSA patients compared with the control and PS groups. After controlling the effects of confounding factors, i.e. age and Body Mass Index (BMI) (which was performed through multiple regression analysis) significant differences were observed among the three groups with regard to N1. However, with regard to SWS, after controlling confounding variables (age and BMI), no significant difference was found among the groups.

**Conclusion:**

The results indicated that OSA, regardless of age and BMI, may increase light (N1) sleep possibly via a decline in blood oxygen saturation (SpO_2 _). Such increase in N1 may be responsible for brain arousal. In addition, by controlling confounding factors (age and BMI), OSA did not affect SWS in OSA patients. However, further research is necessary to determine sleep architecture in more detail in the patients with OSA.

## 1. Introduction

Obstructive Sleep Apnea (OSA) is the most common type of sleep disorder which has increased over the last few decades in developed countries due to the changes in lifestyle along with an increase in the prevalence of obesity (Khazaie et al., 2011; Punjabi, 2008). Based on recent studies on general population, the prevalence of OSA is 3%–7% in adult men and 2%–5% in adult women (Punjabi, 2008).

Sleep-Disordered Breathing (SDB) is recognized as a group of sleep disorders, ranging from Primary Snoring (PS) to Obstructive Sleep Apnea (OSA) (Nisbet, Yiallourou, Walter, & Horne, 2014). PS is a condition with snoring (repetitive sounds due to vibration of upper airway structures) during sleep without apnea or hypopnea (Zhu et al., 2014). OSA is defined as repeated periods of breathing cessation, called apnea or partial obstructions in the upper airway, hypopnea, lasting 10 seconds for the either one (Malhotra & White, 2002). The Apnea-Hypopnea Index (AHI) is defined as the number of apnea and hypopnea events per hour of sleep. An AHI greater than 5 can be diagnosed as OSA (Moon, Punjabi, & Aurora, 2015).

Sleep architecture refers to circular pattern of sleep which changes among different sleep stages, such as non-Rapid Eye Movement (non-REM) and rapid eye movement (Alabi et al., 2012) which normally starts with lighter sleep stages, stage 1 non-REM (N1) and gradually shift to non-REM stages of sleep, each deeper than previous, until it reaches the final stage, Slow Waves Sleep (SWS) stage (Hirshkowitz, 2004). Each sleep cycle eventually leads to REM sleep stage. This non-REM/REM cycle repeats several times until the person wakes up (Smagula et al., 2015).

The most common symptoms of sleep apnea include excessive daytime sleepiness, fatigue, restless sleep, and morning headache. Among these, excessive daytime sleepiness, due to an increased risk of accidents is very serious (Khazaie & Maroufi, 2014). However, daytime sleepiness is absent in 40%–50% of OSA patients (Kapur, Baldwin, Resnick, Gottlieb, & Nieto, 2005; Roure et al., 2008). Therefore, this disorder goes undetected and left untreated. On the other hand, OSA accounts as an alarm for many severe diseases, including hypertension (Torres, Sanchez-de-la-Torre, & Barbe, 2015), cardiovascular diseases (De Torres-Alba et al., 2013; Devulapally, Pongonis, & Khayat, 2009), stroke (Barone & Krieger, 2013), and even diabetes (Cass, Alonso, Islam, & Weller, 2013; Tasali, Mokhlesi, & Van Cauter, 2008). For these reasons, diagnosis and treatment of OSA is necessary.

Today, Polysomnography (PSG) is considered as the most accurate method for diagnosing of OSA (Kapoor & Greenough, 2015). Polysomnography is composed of several different leads to record physiological signals such as Electroencephalogram (EEG), Electroocculogram (EOG), airflow, oxygen saturation, respiratory effort, heart rate and chin electromyogram. Continuous Positive Airway Pressure therapy (CPAP) is the best known and the gold standard treatment for OSA (Spicuzza, Caruso, & Di Maria, 2015; Vlachantoni et al., 2013).

Several studies have indicated that age strongly influences sleep architecture (Ayalon, Ancoli-Israel, & Drummond, 2010; Dorffner, Vitr, & Anderer, 2015; Punjabi, 2008). In general, there is a tendency to increase in light sleep and a decrease in deep sleep with increasing age (Ohayon, Carskadon, Guilleminault, & Vitiello, 2004; Redline et al., 2004; Smagula et al., 2015). Also, there is strong evidence that increased body mass index (BMI) may lead to changes in sleep structure. As previously reported, there is a reverse correlation between BMI and SWS (Rao et al., 2009). There is always a concern in studies that examine the sleep architecture in people with OSA, because the confounding factors, age and BMI, can affect the accuracy of results.

Because of limited information on sleep architecture in the patients with PS and OSA, further investigations are needed to clarify precise sleep architecture in these people. This study aimed to investigate the sleep architecture by controlling age and BMI among the people with OSA, PS and those lacking sleep respiratory disorder. We hypothesized that sleep architecture features are different among these three groups.

## 2. Methods

### 2.1. Study patients

The polysomnographic data used in this study were obtained from 391 clients (from Jan 2012 to Apr 2015) who referred to sleep disorder research center (Kermanshah, Iran). We excluded those people who suffered from diabetes, pulmonary disorders, mental disorders, cardiovascular and neuromuscular diseases, drug users, cigarette smokers and people who had been taking medications affecting sleeping. Subjects were divided into three groups based on their AHI and snoring index: normal group with an AHI<5 without snoring; primary snoring group with AHI<5 with snoring, and, OSA group with AHI ≥5 with or without snoring.

### 2.2. Study procedures

AHI and snoring index was derived from polysomnography taken from subjects. After explaining the procedure and providing sufficient time for them to familiarize with the environment, all subjects underwent a full overnight polysomnography. We analyzed some sleep parameters of sleep architecture such as Total Sleep Time (TST) which is defined as the period of time spent sleeping except wakefulness intervals, the percentage of total sleep time spent in stage 1 of non-REM sleep, the percentage of sleep time spent in stage 2 of non-REM sleep, the percentage of sleep time spent in slow waves sleep (stages 3 and 4) of non-REM sleep, and lastly the percentage of sleep time spent in REM sleep. The minimal arterial oxygen saturation (minimal SpO_2 _), wake index (total number of awakenings per hour of sleep), and brain arousals (total number of brain arousals in the total sleep time) also were evaluated in the groups.

### 2.3. Statistical analyses

The obtained data were initially assessed for normality using Kolmogorov-Smirnov test. Afterwards, the mean (SD) values were used for normally distributed data, median (interquartile range) values for non-normally distributed data, and frequency percentage for categorical data. The Chi-square test was used to determine the differences among the three groups. The differences among the obtained means were analyzed using 1-way ANOVA followed by Tukey test and the differences among the medians were analyzed using Kruskal-Wallis test followed by Dunn’s test. Multiple linear regression analysis was used to adjust confounding variables (BMI and age). All study data were analyzed using SPSS 18 and P values<0.05 was considered statistically significant.

## 3. Results

Table 1 presents the demographic data of 391 study subjects. The mean (SD) age and BMI values (46±14 y and 28.67±4.2 kg/m^2^, respectively) of the subjects of the OSA group were significantly higher than those of the PS and control groups (P<0.001). Therefore, multiple regression analysis was used to control the effects of these two confounding variables (Table 3).

**Table 1.** Demographic data

		**Normal**	**PS**	**OSA**	**P**
Age, y		36.21^a^±14.03	38.82^a^±17.42	46.23^b^±13.9	<0.001^‡^
AHI		1.9^a^(2.75)	2.3^a^(2)	15.7^b^(20.35)	<0.001^†^
BMI, kg/m^2^		24.3a±3.81	25.39^a^±4.85	28.69^b^±4.78	<0.001^‡^
Gender n (%)	Female	24(17.9)	41(30.6)	69(51.5)	
	Male	27(10.5)	60(23.3)	170(66.1)	0.01^§^
Job n (%)	Unemployment	22(19.1)	36(31.3)	57(49.6)	
	Employment	23(11.3)	48(23.6)	132(65.0)	
	Driver	6(10.3)	14(24.1)	38(65.5)	0.077^#^
	Shift worker	0(0.0)	3(20.0)	12(80.0)	
Educational status n (%)	Illiterate	2(5.7)	9(25.7)	24(68.6)	
	Primary	13(14.8)	27(30.7)	48(54.5)	
	High school & diploma	20(15.0)	26(19.5)	87(65.4)	0.32^§^
	Academic	16(11.9)	39(28.9)	80(59.3)	
Marriage statues n (%)	Single	18(22.2)	31(38.3)	32(39.5)	
	Married	27(9.7)	59(21.2)	192(69.1)	0.004^#^
	Widow or divorced	6(18.8)	11(34.4)	15(46.9)	

PS: Primary Snoring; OSA: Obstructive Sleep Apnea; BMI: Body Mass Index; AHI: Apnea–Hypopnea Index

Mean±SD, median (interquartile range), and percentage (%) are presented for parametric, nonparametric, and categorical data, respectively. Means or median with same superscript letters are not significantly different (P>0.05).

†: Kruskal-Wallis test followed by Dunn’s multiple comparisons test was used.

‡: One-way ANOVA test followed by Tukey test was used.

§: The Chi-square test was used.

#: Monte Carlo Chi-square was used.

The results showed an uneven distribution with regard to gender among the groups (P=0.01). The distribution of other variables, such as job and educational status, did not show any significant difference among the groups (P>0.05). However, marital status among the groups had a different and significant distribution (P=0.004) as 69.1 % of the OSA subjects were married (Table 1).

**Figure 2.**
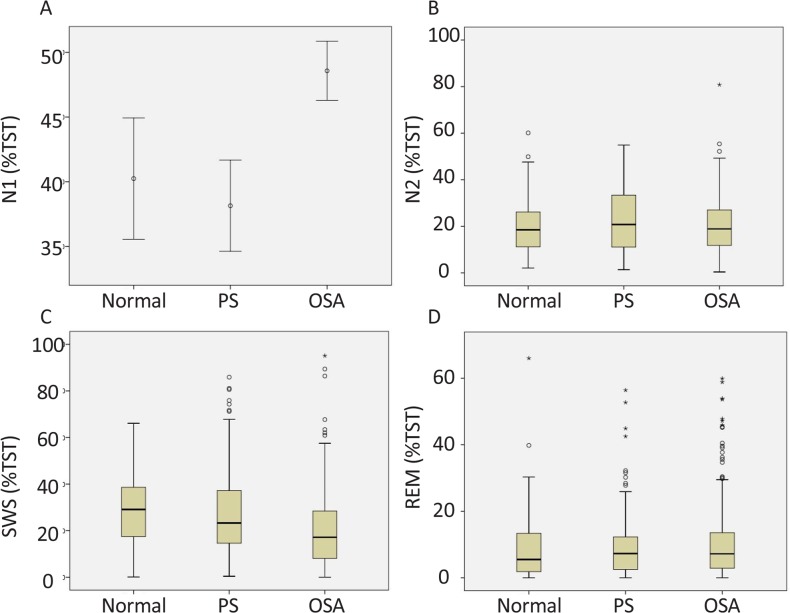
The effect of PS and OSA on sleep architecture (sleep stages) N1 stage showed a significant increase induced by OSA (A) and SWS showed a significant decline induced by OSA (C). N2 (B) and REM (D) stages did not show any difference among the groups. The data of N1 was normally distributed and analyzed using One-way ANOVA test followed by Tukey’s test; but, the data of N2, SWS, and, REM stages were non-normally distributed and analyzed using Kruskal-Wallis test followed by Dunn’s multiple comparisons test. TST: Total Sleep Time; N1, stage 1 of non-REM sleep; N2, stage 2 of non-REM sleep; SWS: Slow-Wave Sleep; REM: Rapid Eye Movement.

With regard to the PSG data (Table 2), the total sleep time in the three groups was more than 6 h (maximum 6.5 h in the OSA group and minimum 6.07 h in the normal group) and no significant difference was found among the groups (P=0.688). Sleep Efficiency (SE) in all three groups was more than 80% and no significant difference was observed among these groups (P=0.647). However, N1 showed a significant difference between the OSA and two other groups; the highest percentage (48.57%) was observed in the OSA group (P<0.001) (Table 2 and Figure 2). With regard to N2, no significant difference was observed among the groups. However, Slow Wave Sleep (SWS) (N3 and N4 stages of non-REM sleep) revealed a significant difference among the groups (P<0.001) as the lowest SWS (17%) was observed in the OSA group and the highest SWS in the normal group (29.1%) (Table 2 and Figure 2). As demonstrated in Table 2 and Figure 2, the percentage of REM sleep of TST showed a similar distribution among three groups (minimum 9.6% in the normal group and maximum 10.65% in the OSA group) and there was no significant difference (P=0.721). As shown in Table 2, minimal SpO_2_ in the OSA group with a mean (SD) of 84(11) indicated a significant reduction compared to those of the other two groups with a mean of 90% (P<0.001). Brain arousal (in TST) showed a significant increase (with a mean [SD] of 27.14 [8.29]) in the OSA group in comparison with the other groups (P<0.001). Wake index in the three groups showed no significant difference among the groups (Table 2).

**Table 2.** Polysomno graphic data

	**Normal (n=51)**	**PS (n=101)**	**OSA (n=239)**	**P**
TST (h)	6.72^a^(1.14)	6.89^a^(1.26)	6.605^a^(1.25)	0.688^†^
SE	88.1^a^(13.37)	89.5^a^(13.2)	87.3^a^(13.68)	0.647^†^
N1 (%TST)	40.24^a^±16.86	38.15^a^±17.84	48.57^b^±17.81	<0.001^‡^
N2 (%TST)	18.5^a^(15.08)	20.8^a^(23.75)	18.9^a^(15.3)	0.37^†^
SWS (%TST)	29.1^b^(21.53)	23.3^b^(25.2)	17^a^(20.5)	<0.001^†^
REM (%TST)	5.5^a^(11.7)	7.3^a^(10.5)	7.2^a^(10.8)	0.721^†^
Minimal SpO_2_	90^b^(4)	90^b^(5)	84^a^(11)	<0.001^†^
Brain arousals (in TST)	23.97^a^±6.38	23.91^a^±7.32	27.14^b^±8.29	<0.001^‡^
Wake index	4.2^a^(4.55)	3.1^a^(5.5)	3.6^a^(4.6)	0.834^†^

PS: Primary Snoring; OSA: Obstructive Sleep Apnea; TST: Total Sleep Time; SE: Sleep Efficiency; N1, stage 1 of non-REM sleep; N2, stage 2 of non-REM sleep; SWS: Slow-Wave Sleep; REM: Rapid Eye Movement; Minimal SpO_2_, minimal oxygen saturation Mean±SD, median (interquartile range), and percentage (%) are presented for parametric, nonparametric, and categorical data, respectively. Means or median with same superscript letters are not significantly different (P>0.05).

†: Kruskal-Wallis test followed by Dunn’s multiple comparisons test was used.

‡: One-way ANOVA test followed by Tukey’s test was used.

**Figure 3.**
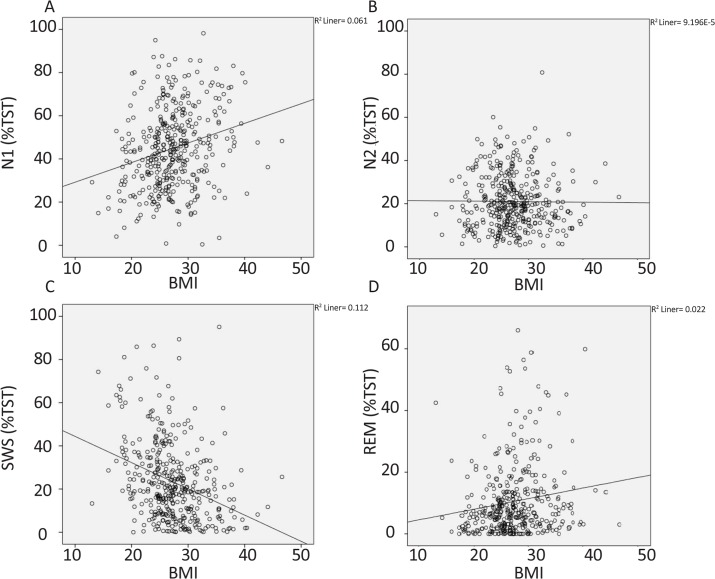
Correlation between BMI and sleep architecture (sleep stages) N1 showed a positive correlation with BMI (A) and SWS showed a negative correlation with BMI (C). N2 (B) and REM (D) did not have any significant correlation with BMI. BMI: Body Mass Index; TST: Total Sleep Time; N1, stage 1 of non-REM sleep; N2, stage 2 of non-REM sleep; SWS: Slow-Wave Sleep; REM: Rapid Eye Movement

Because age and BMI confounding factors were significantly different among the groups (Table 1 and Figure 3), we used multiple regression analysis to control their effects (Table 3). It should be noted that only those variables which were statistically significant in Table 2 (N1 and SWS), were reanalyzed and presented in Table 3 to control the effects of the confounding factors. The analysis indicated a significant difference in observed N1 among the three groups (95%CI=0.42–5.6) (Table 3). In the case of SWS, no significant difference was found among the groups (95%CI=−4.6–0.5) (Table 3).

**Table 3.** Multiple regression model

**Outcome Variable**	**Predictor Variable**	**B**	**95%CI**	**SE**	**P**
N1 (%TST)	Age	0.24	(0.16–0.41)	0.062	<0.001
	BMI	0.108	(0.004–0.78)	0.199	0.048
	Category	0.117	(0.42–5.6)	1.31	0.023
SWS (%TST)	Age	−0.131	(−0.27– −0.33)	0.061	0.013
	BMI	−0.252	(−1.3– −0.54)	0.196	<0.001
	Category	−0.081	(−4.6–0.5)	1.29	0.114

Multiple regression model was used to adjust confounding variables (age and BMI) to determine the significant differences in sleep stages (N1 and SWS) among the groups. Abbreviations: Category, study groups; CI: Confidence Interval; TST: Total Sleep Time; BMI: Body Mass Index; SWS: Slow Wave Sleep; BMI: Body Mass Index

PSG data were also examined in the age ranges of 15–24, 25–44, 45–64, and 65–84 years. As illustrated in Table 4, BMI showed a significant increase in the age range of 15–24 years of the OSA group as compared with the same age range of the PS (P=0.022) group. In addition, in this age range, minimal SpO_2_ demonstrated a significant decrease in the OSA group compared with the control group (P=0.014). As shown in Table 4, in the case of 25–44 age range, BMI indicated a significant increase in the OSA group compared to the two other groups (P=0.002). On the other hand, minimal SpO_2_ showed a significant decrease in OSA compared to the others (P<0.001). Brain arousals showed a significant increase in the OSA group as compared with the PS but with the control group (P=0.001).

**Table 4.** Age classification of variables

	**15–24 Year**				**25–44 Year**			

	**Normal**	**PS**	**OSA**	**P**	**Normal**	**PS**	**OSA**	**P**
AHI	1.3^a^±0.92	2.2^a^±1.16	13.86^b^±18.72	0.011^‡^	2.3^a^(3.3)	2^a^(2.32)	13.6^b^(14.8)	<0.001^†^
BMI	21.63^a^±3.46	21.93^ab^±2.34	24.95^b^±3.92	0.022^‡^	24.99^a^±3.28	25.96^ab^±3.76	27.73^b^±3.79	0.002^‡^
TST (h)	6.97±0.56	7.12±0.67	6.97±0.99	0.849^‡^	6.60±.84	6.42±1.56	6.88±.71	0.072^‡^
SE	91.67±5.70	91.87±5.29	91.52±9.10	0.992^‡^	86.5(21.6)	89.6(12.65)	90.9(11.6)	0.206^†^
N1 (%TST)	35.19±12.05	32.85±8.76	36.54±10.09	0.640^‡^	40.03±17.26	39.98±17.15	44.03±15.13	0.329^‡^
N2 (%TST)	15.53±8.45	22.35±13.93	22.72±7.15	0.149^‡^	22.22±15.08	24.59±13.02	23.74±12.25	0.774^‡^
SWS (%TST)	38.36±15.87	33.63±18.09	32.47±14.39	0.621^‡^	27.98±15.08	24.36±19.06	22.37±15.22	0.355^‡^
REM (%TST)	10.92±18.27	7.69±8.70	8.28±11.44	0.810^‡^	7.1(14.4)	7.85(11.63)	7.3(10.6)	0.922^†^
Minimal SpO_2_	91.92^b^±1.51	90.31^ab^±3.52	88.87^a^±2.17	0.014^‡^	91^b^(4)	91^b^(3)	87^a^(6)	<0.001^†^
Brain arousals (in TST)	25.50±4.17	27.45±5.01	25.80±5.99	0.594^‡^	24.85^ab^±5.38	22.73^a^±7.73	27.88^b^±7.87	0.001^‡^
WI	3.62±2.02	2.20±1.79	2.76±2.97	0.333^‡^	4.3(7)	3.3(5.8)	3.1(3.1)	0.305^†^

Abbreviations: PS, primary snoring; OSA, obstructive sleep apnea; AHI, apnea–hypopnea index; BMI, body mass index; TST, total sleep time; SE, sleep efficiency; N1, stage 1 of non-REM sleep; N2, stage 2 of non-REM sleep; SWS, slow-wave sleep; REM, rapid eye movement; Minimal SpO_2_, minimal oxygen saturation. Mean±SD, median(interquartile range), and percentage(%) are presented for parametric, nonparametric, and categorical data, respectively. Means or median with same superscript letters are not significantly different(P>0.05).

† Kruskal-Wallis test followed by Dunn’s multiple comparisons test was used.

‡ One-way ANOVA test followed by Tukey test was used.

§ Independent Samples t test was used.

**Figure 1.**
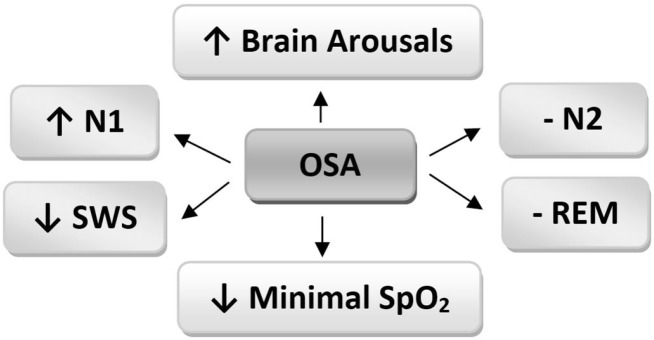
The effects of OSA on sleep architecture ↑: Increase; ↓: Decrease; -: No effect; OSA: Obstructive Sleep Apnea; N1: Stage 1 of Non-REM sleep; N2: Stage 2 of Non-REM sleep; SWS: Slow-Wave Sleep; REM: Rapid Eye Movement; Minimal SpO2: Minimal oxygen saturation

The results obtained from PSG data of 45–64 and 65–84 years age groups are presented in Table 5. According to the results, BMI in the age range of 45–64 years, showed a significant increase in the OSA group compared with the same age range of the control and PS groups (P=0.002). OSA had a significant increase (P=0.003) in N1 and a significant decrease (P=0.021) in SWS at the age range of 45–64 years as compared with the PS; however, it did not demonstrate any significant difference in these variables when compared with the control group. Minimal SpO_2_ in the OSA group was significantly (P<0.001) lower than the other groups. Lastly, brain arousals showed a significant increase in the OSA group as compared with merely the control (P=0.02). Finally, as it has been presented in Table 5, in the 65–84 years age group, only wake index revealed a significant increase in the OSA group compared with the PS group (P=0.001). It should be noted that no subject of this age range (65–84) was in the control group. In brief, as illustrated in Figure 1, OSA increased N1 and brain arousals and decreased SWS and minimal SpO_2_; however, it did not have any effect on N2 or REM stages.

**Table 5.** Age classification of variables

	**45–64 Year**				**65–84 Year**								
	**Normal**	**PS**	**OSA**	**P**	**Normal**	**PS**	**OSA**	**P**
AHI	2.25^a^(3.15)	2.5^a^(1.4)	20.1^b^(27.4)	<0.001^†^	-	2.21±1.43	21.24±14.37	<0.001§
BMI, kg/m^2^	25.71^a^±3.69	28.14^ab^±5.31	29.99^b^±4.93	0.002^‡^	-	25.59±4.25	27.68±4.61	0.312§
TST (h)	6.8(1.13)	6.47(1.45)	6.47(1.31)	0.593^†^	-	5.87±.68	6.27±.61	0.164§
SE	88.1(11.52)	84.9(15.4)	84.5(16.2)	0.37^†^	-	76.49±8.99	83.11±8.73	0.108§
N1 (%TST)	45.65^ab^(18.25)	40.8^a^(29.9)	53^b^(26.35)	0.003^†^	-	56.83±22.99	54.54±18.46	0.799§
N2 (%TST)	21.7(17.95)	21.8(25.5)	17.6(13.3)	0.246^†^	-	11.10±9.05	14.11±7.23	0.398§
SWS (%TST)	21.55^ab^(28.55)	22.2^b^(18.7)	14^a^(18.75)	0.021^†^	-	24.09±22.95	20±15.95	0.621§
REM (%TST)	5.4(9.53)	7.9(11.6)	7.2(11.5)	0.836^†^	-	7.97±9.44	11.33±13.93	0.567§
Minimal SpO_2_	89^b^(4)	87^b^(6)	82^a^(12)	<0.001^†^	-	89±2	77±15	0.53§
Brain arousals (in TST)	21.57^a^±8.60	22.99^ab^±6.65	26.64^b^±8.87	0.02^‡^	-	27.11±10.46	28.62±7.84	0.702§
WI	5.05(4.47)	4.3(7.7)	4.3(5.75)	0.832^†^	-	9.89±4.23	4.75±2.21	0.001§

Abbreviations: PS: Primary Snoring; OSA: Obstructive Sleep Apnea; AHI: Apnea–Hypopnea Index; BMI: Body Mass Index; TST: Total Sleep Time; SE: Sleep Efficiency; N1: stage 1 of Non-REM sleep; N2: stage 2 of Non-REM sleep; SWS: Slow-Wave Sleep; REM: Rapid Eye Movement; Minimal SpO_2_: Minimal Oxygen Saturation

Mean±SD, median (interquartile range), and percentage (%) are presented for parametric, nonparametric, and categorical data, respectively. Means or median with same superscript letters are not significantly different (P>0.05).

†: Kruskal-Wallis test followed by Dunn’s multiple comparisons test was used.

‡: One-way ANOVA test followed by Tukey test was used.

§: Independent Samples t test was used.

## 4. Discussion

The study findings show that people with OSA experience more light sleep and less deep sleep as compared with the people without apnea and even people who are having snoring without apnea (primary snorer). Furthermore, the obtained data indicate that the OSA groups also have a lower minimal SpO_2_ in comparison with the control group. Finally, we found no significant differences in total sleep time and Sleep Efficacy (SE) among the groups studied.

The study results also show that gender is not equally distributed in study groups, so that most people who were diagnosed with OSA were men but women constituted a higher percentage in PS group (P=0.01). Many studies have confirmed our findings about a higher prevalence of OSA in men (Chan, Wong, Tang, & Ng, 2012; Pamidi, Knutson, Ghods, & Mokhlesi, 2011; Punjabi, 2008; Quintana-Gallego et al., 2004; Redline, Kump, Tishler, Browner, & Ferrette, 1994). However, contrary to our findings that demonstrated a higher prevalence of snoring in women, other studies reported that snoring was more prevalent in men (Alabi et al., 2012; Bouscoulet et al., 2008; Chan et al., 2012). Based on the obtained data from clients who were referred to the sleep center, these results cannot be a good representation of the community. In this regard, Zhan and colleagues in a systematic review published in 2012 about gender difference in snoring, reported that a selection bias existed in studies intended to examine the effects of gender in snoring and therefore their results were overshadowed by this issue (Chan et al., 2012).

Furthermore, there was no significant difference between normal and PS group with regard to sleep architecture. In this regard, previous studies have not found any significant difference in sleep architecture of snorers and non-snoring subjects (Fuentes-Pradera et al., 2003; Hoffstein, Mateika, & Mateika, 1991). Hoffman investigated the sleep architecture of heavy snoring and light snoring subjects without any difference in their age, BMI and AHI and didn’t find any significant difference in sleep architecture between these groups (Hoffstein et al., 1991).

The results of our study agree with the previous investigation concerning the effect of age and BMI variables on the sleep architecture. Our findings also show a positive correlation between age and the risk of OSA. Thus, people with OSA have a higher average age compared with people without OSA, as previous studies have reported (Jennum & Riha, 2009; Punjabi, 2008). In this study, we used relevant statistical analysis methods to control age and BMI. In addition, the clients were classified into four age ranges and PSG data in these age ranges were reanalyzed.

The results showed that by controlling the age and BMI effects on the sleep architecture, the percentage of N1 in OSA group was significantly higher than the other two groups. As it was revealed by 1-way ANOVA with Tukey post hoc test, this significant difference was observed only in 45–65 years age group. This finding is consistent with the results of an epidemiological study indicating the majority of OSA patients to be in 50–59 years age range and that a reduction in the risk of OSA is observed after the age 60 (Gislason, Almqvist, Eriksson, Taube, & Boman, 1988). In this regard, other studies have reported an increase in the risk of OSA until the age 60 and a reduction thereafter (Bixler, Vgontzas, TenHave, Tyson, & Kales, 1998; Cass et al., 2013). Therefore, these studies support the validity of our results.

With regard to slow-wave sleep, the initial tests showed a significant difference among three groups. However by controlling age and BMI, as confounding factors, SWS showed no significant difference among the groups. It is difficult to explain these results, but it might be related to the potent effects of confounding variables. Rao et al. (2009) showed a negative correlation between BMI and SWS (Rao et al., 2009). Moreover, some studies have also shown that SWS decreases with increasing age (Ohayon et al., 2004; Redline et al., 2004). Therefore, the effect of confounding factors such as age and BMI could reduce SWS in OSA patients (Ohayon et al., 2004; Rao et al., 2009; Redline et al., 2004; Zhu et al., 2014). In other words, the effect of OSA on reducing SWS may be due to age and BMI variables on sleep architecture.

Also in this study, demographic variables such as employment status, education, and marital status were evaluated. With regard to employment status and education, no statistically significant difference was found among three groups. Regarding marital status of the subjects, most people with OSA were married and such difference can, at least be due to sampling of this study, as the majority of the OSA married subjects were forced by their spouses to refer to our research center.

The sleep architecture of the patients with obstructive sleep apnea showed a tendency to increase light (N1) sleep even by controlling confounders, age, and BMI. It means that, OSA, independent of age and BMI, is able to increase light sleep. However, the effect of OSA on the reduction of deep (SWS) sleep depends on the individual factors such as age and BMI, and OSA independently has no effect on decreasing deep sleep.

It seems that OSA, because of some degree of brain arousal, which is in turn the result of a reduction in arterial blood oxygen concentration, will cause the OSA subjects to spend more time in light sleep and less time in deep sleep. However, in our study this condition apparently has no effect on TST and SE. Perhaps that is why the patients do not complain of insomnia and are sometimes unaware of their problems.

The limitations of this study include: 1. The study subjects were not chosen from general population but from those referred to sleep disorder sleep center from clinics and hospitals; 2. According to the results of epidemiological studies, OSA has the highest prevalence in middle age individuals (Jennum & Riha, 2009; Lee, Nagubadi, Kryger, & Mokhlesi, 2008; Punjabi, 2008; Young, Peppard, & Gottlieb, 2002) and also, in this study, the mean (SD) age of participants was 42.53(15.64) years; and 3. Age and BMI had a heterogeneous distribution among the groups; however, this limitation was resolved by using relevant statistical analysis.

Our study has shown that OSA, independent of age and BMI, might reduce N1 and this reduction can be due to a fall in arterial blood oxygen and a subsequent brain arousal. In addition, by controlling confounding factors; i.e. age and BMI, through performing multiple regression, it was found that OSA has no effect on reducing deep (SWS) sleep in the patients with OSA. However, further research is necessary to determine sleep architecture in patients with OSA.
